# Supplementary material for: Are carbon emissions trading and green financial instruments synergistic? -Comprehensive quantitative research based on content analysis
Source: PLoS One. 2024 Mar 7;19(3):e0298601. doi: 10.1371/journal.pone.0298601 (PMC10919601; doi:10.1371/journal.pone.0298601)
Supplement: S1 Data — (ZIP) [file pone.0298601.s001.zip › Data/地级市Z_F工作报告.zip!/║■▒▒/╛ú╓▌╩╨/└┤╫╘╣1⁄2╓┌║┼í╛┬φ┐╦╩2╛▌═°í┐_2007.docx]

现在，我代表市人民政府向大会作政府工作报告，请予审议，并请市政协各位委员和列席人员提出意见。

过去的一年，在省委、省政府和市委的正确领导下，在市人大、市政协的监督支持下，市政府认真贯彻落实科学发展观，紧紧依靠全市广大干部群众，开拓创新，扎实工作，较好地完成了市三届人大二次会议确定的工作目标和任务，实现了“十一五”良好开局。全市生产总值438.1亿元，增长9.2%，增速比上年提高1.1个百分点；全社会固定资产投资149.7亿元，增长34%；财政收入29.1亿元，增长19%；社会消费品零售总额254.1亿元，增长14.7%；城镇居民人均可支配收入8718元，增长7.7%；农民人均纯收入3502元，增加394元，增长12.7%。

改革开放取得新成绩。农村综合配套改革继续深化，乡镇机构和事业单位改革及国有农场改革任务基本完成，分流乡镇机关人员929人、撤并转事业单位1011家。强化国有资产监督管理，加快非工企业改革步伐，妥善解决职工安置等遗留问题。财政改革进一步深化，财政与编制政务公开全面推进。事业单位改革力度加大，市直减少事业编制2239名。切实加强行政服务中心建设，严肃查处“三乱”行为，规范市场秩序，经济环境不断优化。外向型经济取得新进展。招商引资到位资金75亿元，增长49.5%；实际利用外资8738万美元，增长26%。海关、出入境检验检疫、口岸等大通关建设成效明显，外贸出口突破3亿美元大关，增长27.8%；出口过千万美元的企业达到6家；盐卡口岸集装箱吞吐量突破4万标箱，增长78%。

各项社会事业全面加强。努力提高科技创新能力，国家、省级高新技术企业达到61家，新增11家。优先发展教育事业，认真落实“两免一补”政策，资助贫困学生10.3万人。大力发展职业教育和高等教育，在校大学生达到11.7万人。积极发展文化事业，大型民俗舞蹈诗《呀口火咿嗬》取得较好社会影响，《垄上行》电视栏目受到省表彰，社会科学、文学艺术、新闻出版等取得新成绩。广泛开展群众性体育运动，参加省第12届运动会获团体总分第三名。进一步建立健全公共卫生服务体系，扎实推进新型农村合作医疗制度，加强城市社区卫生服务网络建设，规范医疗服务行为，医疗机构服务收费标准总体下降10.2%。狠抓计划生育基层基础工作，符合政策生育率达96.8%，综合考核为全省第二名。认真清理和规范建设用地，土地资源得到有效保护。加强环境监测和重点污染源整治，加大生态环境建设力度，洪湖37.7万亩拆围任务全面完成。扎实推进“平安荆州”建设，社会治安秩序保持稳定。健全领导包案和部门联动接访机制，妥善解决一批信访突出问题。认真落实安全生产责任制，安全生产工作被评为全省红旗单位。加强国防教育、国防动员、人民防空、民兵和预备役建设，认真做好军转干部、退伍军人安置和拥军优属工作，军政军民关系进一步密切。妇女儿童和未成年人、老年人、残疾人的合法权益得到保护。编制、统计、外事侨务、民族宗教、对台、气象、地震、档案、地方志、质量技术监督、无线电管理等工作都有新进展。

人民生活水平进一步改善。社会保障扩面新增13.3万人次，征缴社保费13.8亿元、增长41.7%。落实退休人员养老提标资金2.35亿元，人月平增资154元。发放救助资金1588.7万元，救助12.5万人；救灾资金870万元、捐赠资金238万元；低保资金1.28亿元，享受低保14万人。坚决纠正损害群众利益的行为，清退教育违规收费645万元，兑现农民工拖欠工资4518万元。

各位代表!过去的一年，成绩来之不易，是省委、省政府和市委正确领导，全市人民团结拼搏，社会各届大力支持的结果。在此，我代表市人民政府，向为荆州改革发展稳定做出积极贡献的广大干部群众，向全市人大代表、政协委员、各民主党派、工商联、人民团体和无党派人士，向人民解放军、武警官兵和政法干警，向所有关心、支持荆州建设与发展的海内外朋友，致以衷心的感谢和崇高的敬意!

二、2007年工作的指导思想和目标

实现上述目标，既有机遇也有挑战。从宏观环境来看，国家实施“中部崛起”战略，省委、省政府高度关心和支持荆州发展，对我们争取政策和资金扶持比较有利。从自身条件来看，实施“工业兴市”战略以来，软硬环境逐步改善，经济发展呈现良好的上升态势。特别是市第三次党代会提出了在中部崛起的大潮中争先进位、开创全面建设小康社会新局面的总要求，为加快发展提供了强有力的思想和组织保证。同时，国家严把土地、信贷“闸门”和市场准入“门槛”，加快发展面临不少压力和挑战。为此，我们务必保持清醒头脑，扬长避短，埋头苦干，努力把各项工作做得更好。

三、促进经济又好又快发展

扎实推进新农村建设。坚持把解决好“三农”问题放在重中之重，加快发展现代农业，着力改善农村生产生活条件，切实增加农民收入，力争农业增加值增长4%。认真落实农村各项政策。严格执行国家支农惠农和粮食最低收购价政策，及时兑现直补资金，引导农村土地经营权有序流转，保护和调动农民生产积极性。加强对农民负担和“一事一议”的监督管理，积极探索化解乡村债务的新途径，防止农民负担反弹。大力发展现代农业。坚持不懈地调整农业结构，加强专业小区和板块建设，积极推广生态、健康种养方式，促进规模化、标准化生产，加快发展具有竞争优势的产业，力争粮食总产增加1亿斤，新增标志性农产品80个以上。坚持不懈地推进农业产业化经营，落实农产品加工企业税收优惠政策，做大做强龙头企业，力争新增市级以上龙头企业20家。积极发展农民专业合作组织，抓好7个省部级试点合作组织建设，每个县市区培育1-2个运作规范、带动力强的农业合作社，搞活农产品流通。坚持不懈地加强基层农业技术推广队伍建设，努力减少动植物疫病损失。积极发展农业机械化，力争机耕、机整、机收面积达到50%以上。加强农村基础设施建设。继续实施荆南四河综合治理、荆江洪湖分蓄洪区、荆江河势控制、引江济汉、洈水水库整险加固等工程，抓好6个大型灌区续建和18处大型泵站更新改造工程建设，扎实做好防汛抗灾工作。认真抓好高产农田建设，大力实施血防整县推进和四湖流域水利血防综合治理工程，加大农村安全饮水工程和通乡通村公路建设力度，不断提高农业综合生产能力。发展壮大县域经济。坚持走“一主三化”的发展道路，用足用好扶持政策，在营造良好环境、培育特色产业上下功夫，推动县域经济争先发展。抓好新农村建设试点。从群众最关心、最迫切、最容易见效的事情抓起，因地制宜，分类指导，整合项目，部门联动，集中力量办好李埠、花基台等139个新农村建设试点。积极发展文化旅游等现代服务业。坚持用市场化的思路、产业化的办法，加快资源整合和开发步伐，努力提高服务业发展水平。加快发展文化旅游业。充分发掘独特的历史文化底蕴和良好的生态资源，把文化旅游产业作为支柱产业来培育，作为战略产业来扶持，重点抓好熊家冢楚墓抢救性发掘与保护利用、纪南城遗址公园和海子湖旅游目的地项目推介及招商建设，积极推动荆州古城整体策划开发，精心打造“楚文化游”、“三国文化游”品牌。加强洪湖、天鹅洲湿地保护和利用，开展洈水4A级景区创建工作，完善瞿家湾、周老嘴红色旅游和黄山头等景区基础设施建设，不断提高旅游开发和管理水平。做大做强商贸流通业。发挥老商埠优势，加强以北京路、中山路、沙隆达广场为骨架的中心商务区建设，加快沃尔玛项目建设进度，进一步推进商业布局和经营业态调整。加强工业品、农产品、消费品和农资等物流规划和设施建设，支持两湖平原农产品交易中心等项目建设，提升荆州商业集聚、辐射功能。加快发展网络通信、信息咨询、中介、社区服务等新型服务业，进一步提高城市综合服务水平。抓好“万村千乡”市场工程，扩大农村市场消费。同时，加强烟草、生猪屠宰和成品油市场管理，加大对食品、药品、餐饮卫生等监管力度，整顿和规范市场秩序。

着力提高城镇化水平。坚持规划先行，突出特色，科学布局，完善功能，增强中心城区核心竞争力，强化其龙头带动作用，提高城镇化水平。加强中心城区建设和管理。进一步加强规划管理，抓紧完成城市总规上报审批工作，编制好荆州工业新区发展规划，加强城市水面、主干道、城乡结合部等区域规划控制，严肃查处违规建设行为，维护规划的权威性。进一步完善城市基础设施，充分发挥市城投公司融资平台作用，积极筹措建设资金，重点实施雨台路、荆沙大道等建设工程，启动城南、草市污水处理厂建设，全面整治护城河、庙湖水体污染。进一步加强城市管理，充分发挥街道、社区在城市管理中的作用，深入实施城市绿化、净化、美化、亮化工程，突出整治占道经营和“脏乱差”，强化环境卫生管理责任和措施，争创全国卫生城市。调整商品房开发结构，加快危旧房改造步伐，扩大经济适用房比重，逐步改善低收入家庭住房条件。加强县市城区和中心镇建设。强化规划引导，拓宽融资渠道，抓好县市城区道路、污水处理等基础设施建设，提高发展内涵和质量。抓紧编制10个重点镇控制性详规，积极推进“百镇千村”示范工程，增强小城镇发展活力。抓好重大基础设施项目建设。紧紧围绕国家、省实施新农村建设和中部崛起战略的相关政策，积极争取项目投资。重点实施沪汉蓉高速客运铁路荆州段、随岳高速公路荆州段、荆岳长江公路大桥、荆州至监利一级公路、江南一级公路、东荆河大桥、盐卡二期多用途码头、石首港工业综合码头等项目，争取盐卡口岸升级为国家一类水运口岸。继续抓好荆岳铁路、荆州港旅游码头等项目前期工作。加强项目协调服务，加快项目建设进度，确保完成年度投资计划。

加强节能降耗和环境保护。坚持把节能降耗、集约用地和保护环境作为基础性工作来抓，促进经济增长方式转变和产业结构优化升级。加强节能降耗工作。重点抓好冶金、化工、建材等高耗能行业和企业的能耗监管，推广应用余热余压回收利用、设备保温节能等技术和产品，淘汰落后工艺、设备和生产能力。认真执行建筑节能设计标准，积极发展节能省地型建筑。增强全社会的节约意识，加强节电、节水等工作。采取产业链条延伸、资源综合利用、废弃物集中处理、热电联供等方式，大力发展循环经济，积极推行清洁生产，提高资源利用效率。加强土地管理。认真执行土地利用总体规划和年度计划，保障重点项目建设用地需要。加强土地开发和整理工作，努力做到占补平衡。加强土地市场建设，完善土地招拍挂制度。严格依法管地用地，提高土地使用效率和集约化程度。加强生态环境保护。牢固树立环保先行的观念，全面推行排污许可、总量控制、限期治理、环境影响评价等制度，坚决关闭严重污染环境的小化工、小纸厂，有效削减主要污染物排放总量。加强生态环境建设，重点实施城市污水和总干渠、西干渠流域污水等整治工程，积极解决群众反映强烈的饮食业油烟、生产生活噪声污染等问题。推广生态农业模式，减少农村面源污染。落实环境保护目标责任制，加大环境执法力度，提高环境监管水平。

全面发展社会事业。增加科技投入，完善激励政策，推动科技创新和科技进步。巩固和提高农村义务教育水平，确保通过国家“两基”工作检查验收。从今年春季开始，免除农村义务教育阶段学生学杂费，对贫困家庭学生免费提供教科书并补助寄宿生生活费，提高农村义务教育阶段中小学预算内公用经费补贴标准。进一步规范中考招生行为，促进教育公平。大力发展职业教育和高等教育，支持每个县市区建设1所设施完备的职教中心，支持长江大学创建高水平综合大学，规范市属高校办学行为，努力提高办学水平。加强公共卫生和医疗服务体系建设，全面推进新型农村合作医疗试点工作，完善社区卫生服务网络，切实做好血吸虫病、艾滋病、结核病等防治工作。继续开展医院管理年活动，推进药品网上集中招标采购，规范医疗机构服务行为，减轻群众医疗费用负担。加强公共文化基础设施建设，扶持建设一批乡镇综合文化站，抓好村级活动场所及“一室多用”的配套设施建设，活跃群众文化生活。加强历史文化遗产保护，扶持发展文博事业。整合有线电视网络资源，加快发展广播电视事业和产业。广泛开展全民健身运动，培育体育后备人才。牢固树立人才资源是第一资源的观念，在培养人才、引进人才、用好人才上下功夫，切实抓好党政人才、企业经营管理人才、专业技术人才及技能型人才队伍建设。加强流动人口计划生育管理和出生人口性别比综合治理，稳定低生育水平。认真做好第二次农业普查工作。积极发展残疾人事业，支持妇女儿童、老年人事业发展，做好民族宗教、对台、外事侨务、档案、地方志、地震等工作。 深入开展精神文明创建活动。大力弘扬以爱国主义为核心的民族精神和以改革创新为核心的时代精神，开展以“八荣八耻”为主要内容的社会主义荣辱观教育，重视和加强未成年人思想道德建设，努力提高公民整体素质和社会文明程度。积极推进文化、科技、法律、卫生“四下乡”、“四进社区”工作，广泛开展文明城市、文明村镇、文明单位创建活动。坚决扫除“黄赌毒”等社会丑恶现象，树立良好的社会风尚。加强基层民主政治建设，充分发挥农村和城镇社区自治组织在社会管理中的重要作用。继续实施中心城区社区管理设施建设三年计划，逐步完善社区功能，不断提高社区管理和服务水平。

认真抓好治安、信访、安全生产等工作。加强社会治安综合治理，严厉打击各种违法犯罪行为，着力整治突出的治安问题，积极创建“平安荆州”。深入开展“五五”普法，做好法律援助和司法救助工作，促进司法公正。认真落实信访工作责任制，努力把矛盾化解在基层。健全应急管理机制，有效应对突发公共事件。高度重视安全生产，严格交通运输、工矿企业、建筑施工等领域安全管理，防止发生重特大安全事故。同时，继续支持驻荆部队建设，进一步加强国防教育、国防动员、民兵、预备役建设和人防工作，积极做好军转复退军人优抚安置工作，争创省级双拥模范城市。

五、加强政府自身建设

坚持依法行政。认真贯彻人大及其常委会的决议，自觉接受法律监督和工作监督，自觉接受政协的民主监督，接受新闻舆论和社会公众监督。认真办理人大代表建议、批评、意见和政协委员提案，广泛听取民主党派、工商联、人民团体和无党派人士的意见。加强行政执法队伍建设，规范执法程序，强化执法监督，严格行政执法责任制和过错追究制，做好行政复议和行政应诉工作，提高依法行政水平。认真执行重点企业保护制度，严肃查处行政不作为、乱作为和损害经济发展环境的行为，维护企业合法权益。

坚持廉洁从政。加强公务员特别是领导干部理想信念和廉洁从政教育，强化宗旨意识和服务意识，努力提高公务员队伍整体素质。围绕规范行政权力运行，推进体制改革和制度建设，切实从源头上治理腐败。认真落实廉政建设责任制，加强行政监察和审计监督，抓好商业贿赂专项治理，坚决纠正损害群众利益的不正之风，严肃查处违纪违规行为。
